# Frailty and pre-frailty associated with long-term diminished physical performance and quality of life in breast cancer and hematopoietic cell transplant survivors

**DOI:** 10.18632/aging.206109

**Published:** 2024-09-26

**Authors:** Najla El Jurdi, Hok Sreng Te, Qing Cao, Char Napurski, Shuo Wang, Andre Robinson, Mukta Arora, Heba ElHusseini, Fiona He, Laura J. Niedernhofer, Bharat Thyagarajan, Anna Prizment, Shernan Holtan, Anne Hudson Blaes, Matthew J. Yousefzadeh

**Affiliations:** 1 Blood and Marrow Transplant Program, Departments of Medicine and Pediatrics, University of Minnesota, Minneapolis, MN 55454, USA; 2 Division of Hematology, Oncology, And Transplantation, Department of Medicine, University of Minnesota, Minneapolis, MN 55454, USA; 3 Masonic Cancer Center, University of Minnesota, Minneapolis, MN 55454, USA; 4 Department of Laboratory Medicine and Pathology, University of Minnesota, Minneapolis, MN 55454, USA; 5 Amgen, Thousand Oaks, CA 91320, USA; 6 Institute on the Biology of Aging and Metabolism, Department of Biochemistry, Molecular Biology and Biophysics, University of Minnesota, Minneapolis, MN 55454, USA; 7 Columbia Center for Translational Immunology and Columbia Center for Healthy Longevity, Department of Medicine, Columbia University Medical Center, New York, NY 10032, USA

**Keywords:** hematopoietic cell transplantation, breast cancer, aging, cellular senescence, frailty

## Abstract

Physical frailty as a sign of accelerated aging is not well characterized in breast cancer (BC) and hematopoietic cell transplant (HCT) survivors and its correlation with outcomes and quality of life (QOL) is not defined. We conducted a prospective study to determine the prevalence of frailty in adult BC and HCT survivors, examine its impact on QOL, and determine its association with *p16^INK4a^*, a molecular biomarker for biological aging. The study included 59 BC and 65 HCT survivors. Median age was 60 years (range 27-81), 68.5% were female and 49.2% were 18-59 vs. 51.8% ≥60 years old. A total of 71 (57.3%) were “fit” (frailty score 0) vs. 53 (42.7%) were pre-frailty/frail (frailty scores ≥1), and of the latter 17 (32.1%) were BC and 36 (67.9%) HCT patients. On multivariate analysis, patients >60 years were twice as likely to be frail (OR 2.04, 95% CI, 0.96-4.33; p=0.07), HCT were more likely to be frail compared to BC patients, and female HCT had 2.43 (95% CI, 0.92-6.40) and male HCT patients had 3.25 (95% CI, 1.37-7.72) times higher risk of frail; p=0.02. Frailty was associated with significant decline in QOL, measured by Medical Outcomes Study (MOS) Short Form 36 (SF-36) Physical Component Summary (PCS) and Mental Component Summary (MCS), and FACT (Functional Assessment of Cancer Therapy) scores. *p16^INK4a^* expression was higher in those who were frail, older than 60, and with higher expression in frail vs. fit patients who are 18-59 years. Our study highlights the high prevalence of frailty in survivors with detrimental effects on physical and overall wellbeing, and supports an association between frailty and the senescence marker *p16^INK4a^*.

## INTRODUCTION

The primary treatment goal in patients with cancer is to “cure” the underlying disease. Recent advances in treatment strategies and supportive care resulted in improved survival in patients with breast cancer (BC), as well as those with hematologic malignancies, particularly those treated with allogeneic hematopoietic cell transplant (HCT). However, cure or remission of the underlying malignancy and surviving is not always accompanied by a full restoration of health. Therefore, ideally, the ultimate goal is to achieve remission with minimal toxicities, without adding morbidities, late effects and/or compromising quality of life (QOL).

Frailty and pre-frailty are debilitating health conditions affecting community dwelling elderly (> 65-years) with a prevalence of 10% [1, 2]. Pre-frailty doubles the risk of frailty compared to fit individuals, which increases mortality, functional decline, health care utilization, and poor quality of life [2–4]. Survivors after cancer treatment with chemotherapy and/or and HCT have been shown to have a higher prevalence of physiologic frailty as compared to sibling controls [5–8]. Additionally, these studies have shown a higher prevalence of frailty in young (< 65 years) survivors, with similar rates to community dwelling elderly, and a higher mortality in frail survivors [7, 8].

Cells experiencing stress can adopt various cell fates, one of them being cellular senescence, where a cell becomes stably arrested in the cell cycle, even in the presence of growth signals. Senescent cells (SnCs) accumulate in the body with age and disease, adversely affecting both healthspan and lifespan [9]. Despite their loss of proliferative capacity, SnCs remain metabolically active and resistant to apoptosis. One distinctive feature of SnCs is their ability to secrete a variety of inflammatory factors that are capable of disrupting tissue homeostasis and contributing to the age-related increase in chronic inflammation known as inflammaging [10]. Quantification of SnC burden in peripheral blood, through the measurement of the senescence marker *p16^INK4a^*, is a useful molecular biomarker for biological aging [11].

While physical frailty as a sign of accelerated aging is recognized in breast cancer and HCT survivors [12, 13], it is not well characterized, and its correlation with outcomes and QOL is not defined. Here, we aim to determine the prevalence of frailty and pre-frailty in adult BC and HCT survivors, examine its impact on functional decline and QOL, and determine its association with molecular biomarkers of accelerated aging.

## MATERIALS AND METHODS

### Study design and inclusion criteria

This is a prospectively accruing observational study conducted at the University of Minnesota transplantation and breast cancer programs from December 2019 through May 2023. Inclusion criteria were: adults (≥18 years old) at the time of BC treatment or HCT, ≥1 year survivors of HCT for any underlying diagnosis or BC treated only with chemotherapy, in complete remission from the underlying malignancy at the time of enrollment.

The primary objective was to determine the prevalence of frailty and pre-frailty in BC and HCT patients surviving more than one year after treatment and compare that in young (18-59) vs. older (≥60 years) survivors. The secondary objectives were to determine the association of: 1. frailty with the continuous summary performance score (CSPS) as a measure of physical functional performance; 2. expression of *p16^INK4a^* in CD3^+^ peripheral blood mononuclear cells (PBMCs), a surrogate aging biomarker, with biological frailty; 3. frailty with QOL as measured by Medical Outcomes Study (MOS) Short Form 36 (SF-36) Physical Component Summary (PCS) and Mental Component Summary (MCS), and FACT (Functional Assessment of Cancer Therapy) scores.

Frailty was defined per Fried’s criteria as a clinical phenotype with > 3 of the following features: unintentional weight loss, exhaustion, slow walking speed, low physical activity and weakness, while 1 or 2 features indicates pre-frailty and non-frail or “fit” indicating the absence of such measures [2]. Physical functional performance was assessed by Karnofsky performance scale (KPS) [14], and continuous summary performance score (CSPS) which is calculated by adding scores from three tests: walking speed, standing balance, and repeated chair stands tests [15].

The SF-36 is a 36-item general assessment of health-related quality of life with eight subscales: Physical Functioning, Role Physical, Pain Index, General Health Perceptions, Vitality, Social Functioning, Role Emotional, and Mental Health Index [16]. Higher scores indicate better QOL. A clinically meaningful difference is 0.5 times the standard deviation, thus a 5-point difference is considered to have clinical significance [17]. The FACT version 4.0 instrument is a 37-item scale comprised of a general core questionnaire, the FACT-G evaluates the health-related QOL of patients receiving treatment for cancer, and specific modules for BMT (FACT-BMT) or breast cancer (FACT-B) Concerns address disease and treatment-related questions specific to BMT or breast cancer. The FACT-G consists of four subscales developed and normed in cancer patients: Physical Well-being, Social/Family Well-being, Emotional Well-being, and Functional Well-being. Each subscale is positively scored, with higher scores indicating better functioning [18, 19]. Expression of *p16^INK4a^*, a marker for cellular senescence, was measured from in CD3^+^ PBMCs as detailed below [11].

All patients signed a written informed consent for the use of their medical data in clinical research. This study was reviewed and approved by the University of Minnesota Institutional Review Board. Data were retrieved from the study specific REDCap database UMN BMT database with supplemental clinical data extracted from the electronic health record.

### Plasma and PBMC isolation

Plasma samples are isolated by centrifuging vacutainer tubes at 1000 x *g* for 10 min at room temperature with the brake set to low. Plasma is pipetted off, aliquoted, and stored at -80° C. Blood samples are resuspended with a volume of 1X DPBS that is equivalent to the volume of plasma removed. Peripheral blood mononuclear cells (PBMCs) were isolated via density gradient separation. Blood samples were first diluted 1:1 with 1X DPBS before being added over a layer of 15 mL of Ficoll-Paque (GE Healthcare) in a 50 mL conical tube. The samples are centrifuged at 750 x *g* for 30 min at room temperature with no brake. After centrifugation the residual plasma layer is pipetted off to allow access to the buffy coat which contains the PBMCs. This is pipetted and transferred to a new 50 mL conical and washed with RPMI 1640 media (containing 10% human AB serum). Samples are then centrifuged vacutainer tubes at 550 x *g* for 10 min at room temperature with the brake set to low. PBMCs are then resuspended in media and cell counting is performed.

### CD3^+^ PBMC isolation

CD3^+^ PBMCs are isolated via magnetic bead purification through negative selection using the EasySep Human T Cell Isolation Kit (StemCell Technologies, Vancouver, Canada). ~10^6^-10^7^ PBMCs are resuspended in the aforementioned media in a flow cytometry tube and incubated with the Human T Cell Isolation Cocktail for 5 min before incubation with the RapidSpheres for an additional minute. The samples are then placed on the EasySep Magnet and left to incubate for 10 min. The supernatant containing unbound CD3^+^ PBMCs can be pipetted off into another tube allowing for cell counting, aliquoting and eventual storage at -80° C.

### Senescence marker measurement by qPCR

Total RNA was isolated from cell pellets using the PureLink RNA Mini kit (Thermo Fisher). cDNA synthesis reactions were performed using 200 ng of total RNA with the High-Capacity cDNA Reverse Transcription Kit with Rnase inhibitor (Thermo Fisher). Expression of *CDKN2A*/*p16^INK4a^* and *18S* RNA was quantified with Taqman qPCR using a QuantStudio 3 real time thermocycler (Thermo Fisher). Expression of *p16^INK4a^* was normalized by determination of ∆Ct^-1^. Probe information for as described in: *18S* (Taqman ID Hs03003631_g1); *p16^INK4a^* (Taqman Custom Assay ID APWCZMM) [11].

### Statistical methods

Descriptive statistics, including mean, median, and range, were employed to characterize demographic, clinical, laboratory, and quality of life measurements variables across the two frailty groups (fit and frail). Categorical variable comparisons between groups were conducted using chi-square test, and continuous variable comparisons between groups were conducted using either Student *t*-tests or Wilcoxon rank-sum tests depending on the distribution. In Table 1, we also reported the p-values comparing between the two frailty groups adjusted by disease group (BC and HCT). Logistic regression was used for categorical variables and linear regression (appropriate log transformation was used for non-normal distributed variables) for continuous variables. Spearman correlation was used to assess the association between continuous variables and normalized *p16^INK4a^* expression in CD3^+^ PBMCs. Multivariate logistic regression analyses were employed to investigate the risk factor of disease associated with the presence of frailty (frailty score ≥ 1) adjusted by age group (18-59 vs. ≥ 60).

**Table 1:** Patient and frailty characteristics.

	**All Groups**	**Fit**	**Frail**	**p-value**	**Adjusted p-value**
	N=124	N=71	N=53		
**Age at Enrollment^†^**				<0.01	<0.01
Median (Min-Max)	60 (27-81)	56. (27 -79)	63 (40-81)		
Age groups at Enrollment^*^				0.03	0.06
18-59	61 (49.2%)	41 (57.7%)	20 (37.7%)		
>=60	63 (50.8%)	30 (42.3%)	33 (62.3%)		
**Age at chemotherapy/Transplant^†^**				<0.01	<0.01
Median (Min-Max)	54 (25-76)	50 (25 -72)	58.5 (32-76)		
**Years from Treatment to Study Enrollment^†^**				<0.01	0.13
Median (Min-Max)	5.0 (1.0-14.8)	5.8 (1.0-14.8)	3.4 (1.0-13.0)		
Group/Diagnosis^*^				<0.01	--
Transplantation	65 (52.4%)	29 (40.8%)	36 (67.9%)		
Breast Cancer	59 (47.6%)	42 (59.2%)	17 (32.1%)		
Gender^*^				<0.01	0.48
Female	85 (68.5%)	55 (77.5%)	30 (56.6%)		
Male	39 (31.5%)	16 (22.5%)	23 (43.4%)		
**BMI at Enrollment^#^**				0.34	0.39
Median (Min-Max)	27.0 (17.6-52.5)	26.5 (19.2-45.3)	27.3 (17.6-52.5)		
**Unintentional Weight LOSS (%)^#^**				0.37	0.55
Median (Min-Max)	-0.5 (-35.3-28.2)	0.000 (-20.3-11.2)	-1.0 (-35.3-28.2)		
Karnofsky Performance Status at Enrollment^*^				<0.01	<0.01
100	54 (43.5%)	47 (66.2%)	7 (13.2%)		
80-90	65 (52.4%)	24 (33.8%)	41 (77.4%)		
≤70	5 (4.0%)	0	5 (9.4%)		
**Total CSPS by adding the scores of the 3 tests^#^**				<0.01	0.01
N of Observed	118	70	48		
Median (Min-Max)	2.56 (0.81-2.91)	2.60 (2.21-2.78)	2.50 (0.81-2.91)		
N of Missing	6	1	5		
**Frailty Domains**					
Weight Loss^*^				<0.01	0.97
1	10 (8.7%)	0	10 (18.9%)		
Exhaustion^*^				<0.01	0.95
1	26 (21.0%)	0	26 (49.1%)		
Physical Activity^*^				<0.01	0.96
1	18 (14.5%)	0	18 (34.0%)		
Walk Time^*^				<0.01	0.97
1	9 (7.3%)	0	9 (17.0%)		
Grip Strength^*^				<0.01	0.94
1	35 (28.2%)	0	35 (66.0%)		

^*^p-values on categorical variables were from Chi-square test excluding missing values, and adjusted p-values by disease group were from logistic regression.

^†^p-values on normally distributed continuous variables excluding missing values were from Student *t*-tests, and adjusted p-values by disease group were from linear regression.

^#^p-values on non-normally distributed continuous variables were from Wilcoxon Rank-Sum test excluding missing values, and adjusted p-values by disease group were from linear regression after appropriate log-transformation. During the log-transformation process, in order to prevent the calculation of logarithms for zero or negative numbers, we applied adjustments to the following variables: 50 was added to Unintentional Weight Loss (%), 1 was added to Chair Stands Test, Physical Functioning, Role Limitations Due to Physical Health, Role.

All statistical tests were two-sided, and significance was established at p < 0.05. The statistical analyses were conducted using SAS 9.4 (SAS Institute, Inc., Cary, NC, USA) and R version 4.2.2 (R Foundation for Statistical Computing, Vienna, Austria).

Frailty is conventionally classified into three groups: fit (score = 0), pre-frail (score of 1 or 2), and frail (score ≥ 3). Due to the limited representation of patients with a frailty score ≥ 3 within the breast cancer study population (only 1 patient), for the purpose of comparative analysis, we will compare two groups of survivors, those with a frailty score of 0 as the “fit” group and those with a score ≥ 1 as the “frail” group.

SF-36 was scored in eight subscales listed above using RAND method [20]. The PCS and MCS are summary scores normalized to a T score of 50 with a standard deviation of 10. The Functional Assessment of Cancer Therapy - Breast Cancer (FACT-B, v4) and the scores of the Functional Assessment of Cancer Therapy - Bone Marrow Transplant (FACT-BMT, v4) were generated by the FACTscorer R package, which was used to calculate the FACT scores from Breast cancer and BMT based on scoring provided by FACIT website (https://www.facit.org/).

### Data availability

All data generated or analyzed during this study are included in this published article. There are no data available or eligible to be made accessible.

## RESULTS

### Patient characteristics and frailty prevalence

The study population included a total of 124 patients, 59 (47.6%) BC and 65 (52.4%) HCT survivors. Median age at the time of enrollment was 60 years (range 27-81), of those 68.5% were female and 49.2% were 18-59 vs. 51.8% ≥60 years old. Median age at time of initiation of treatment (chemotherapy or HCT) was 54 years (25-76), and median time from treatment to enrollment on this study was 5.0 years (1.0-14.8 years). All breast cancer patients were female, while 26 (40%) of HCT patients were female. Median age at treatment was 51 vs. 65 years, and the median age of enrollment was 59 for the BC cohort vs. 65 years in HCT cohort, respectively.

Of all the survivors enrolled, 71 (57.3%) were “fit” with a frailty score of 0 vs. 53 (42.7%) were pre-frailty/frail (“frail” group) with frailty scores ≥1, and of the latter 17 (32.1%) were BC and 36 (67.9%) HCT patients. Of note, only one BC patient had frailty score ≥3, while for HCT patients, 29 had a score of 0, 23 score 1-2 and 13 score ≥3.

### Frailty characteristics and association with outcomes

Table 1 shows the patient characteristics per frailty groups. Frail patients were older with median age 63 (40-81) vs. 56 (27-79) years at the time of study enrollment and older at the time of treatment initiation (adjusted p <0.01). Patients with higher frailty scores were typically older than 60 vs. 18-59 years (62.3% vs. 37.7%) as expected, although this was not significant after adjustment for diagnosis (p=0.03, adjusted 0.06). Frail patients were mostly BMT survivors (67.9% vs. 32.1%, p<0.01). When comparing the fit vs. frail group, we noted no significant difference in baseline body mass index (BMI) at enrollment or weight loss over 1 year prior to enrollment. Physical functional performance was worse in the frail group with KPS groups of 100, 80-90, and ≤70 in 13.2%, 77.4% and 9.4% of frail patients compared to 66.2%, 33.8% and 0% in the fit patients (adjusted p<0.01). Similarly, the CSPS physical functional performance assessment indicated a significantly lower score in the frail compared to the fit group with a median score of 2.50 (0.81-2.91) vs. 2.60 (2.21-2.78), respectively (adjusted p=0.01).

We next examined the association of frailty with QOL as measured by SF-36 and FACT. Frailty was associated with significant decline in QOL as measured by the following six SF-36 subscales: physical functioning (adjusted p<0.01), pain (adjusted p<0.01), energy/vitality (adjusted p<0.01), social functioning (adjusted p<0.01), emotional wellbeing (adjusted p=0.01), and general health perceptions (adjusted p=0.03). The PCS and MCS summary scores were also significantly higher in fit compared to frail patients, with median scores of 46 vs. 36 (adjusted p<0.01) and 53 vs. 46 (adjusted p=0.05), respectively. FACT-G score was significantly higher in fit compared to frail patients in the overall cohort, median score of 92 vs. 75 (adjusted p<0.01). In the BMT cohort, FACT-BMT score was significantly higher at 115 in fit compared to 99 in frail patients, and similarly the FACT-B score in the breast cancer cohort was 123 in fit and 105 in frail patients, adjusted p<0.01 and 0.03, respectively.

Table 2 shows the multivariate logistic regression analysis of disease and age in association with frailty. Patients older than 60 years were twice as likely to be clinically frail, OR (odds ratio) 2.04 (95% CI, confidence interval, 0.96-4.33; p=0.07). HCT recipients were significantly more likely to be frail compared to breast cancer patients, after adjusting for age. Specifically, female HCT recipients had 2.43 (95% CI, 0.92-6.40) times higher risk, and male HCT patients had 3.25 (95% CI, 1.37-7.72) times higher risk of being frail; p=0.02.

**Table 2:** Multivariate logistic regression by frailty group.

Factor*	**N**	**Frailty score ≥1 odds ratio (95% confidence interval)**	**P-value**
**Disease Gender group**			0.02
Breast cancer Female	59	1.00 (reference)	
Transplant Female	26	2.43 (0.92-6.40)	
HCT Male	39	3.25 (1.37-7.72)	
			
**Age**			
age: 18-59	61	1.00 (reference)	0.07
age: >=60	63	2.04 (0.96-4.33)	

*KPS was not included due to significant correlation with disease gender group and age group.

### Frailty association with CD3^+^ expression of *p16^INK4a^*, a biomarker of aging

**Figure 1:**
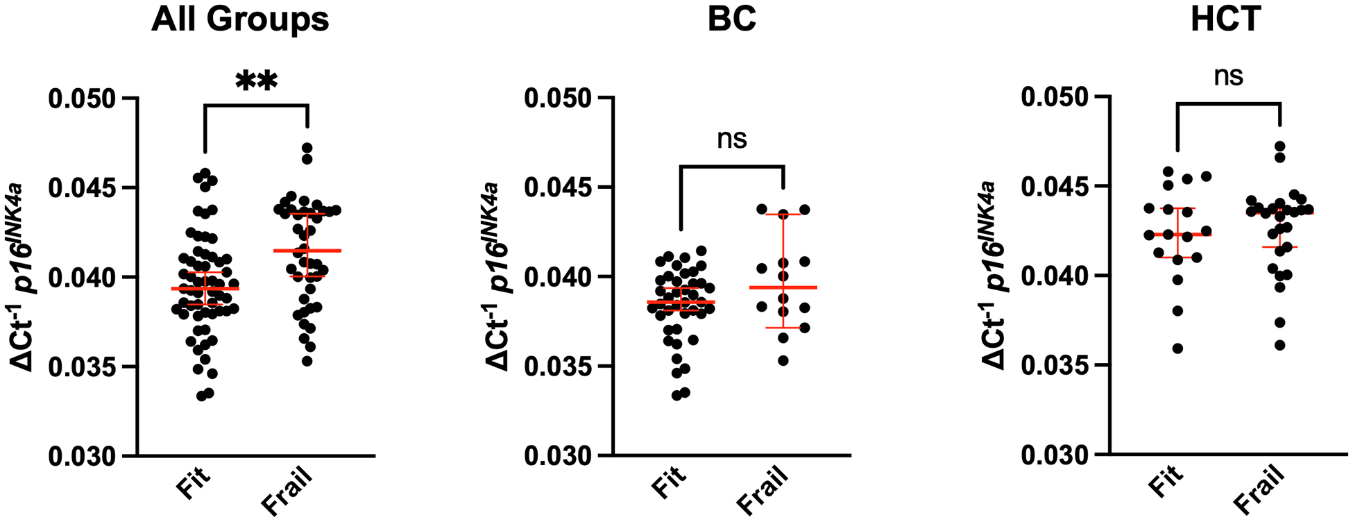
**Comparison of senescence with frailty in breast cancer and bone marrow transplant survivors.** Whole blood from breast cancer (BC) survivors or hematologic malignancy patients treated with allogeneic hematopoietic cell transplantation (HCT) was used to CD3+ peripheral blood mononuclear cells (PBMCs) by magnetic bead purification. Total RNA was used to quantify expression of the cellular senescence marker *p16^INK4a^* by qPCR. Expression was normalized to 18S. Values represent the median with 95% confidence interval. Students unpaired two-tailed *t* test. ** p<0.01, ns (not significant).

**Figure 2:**
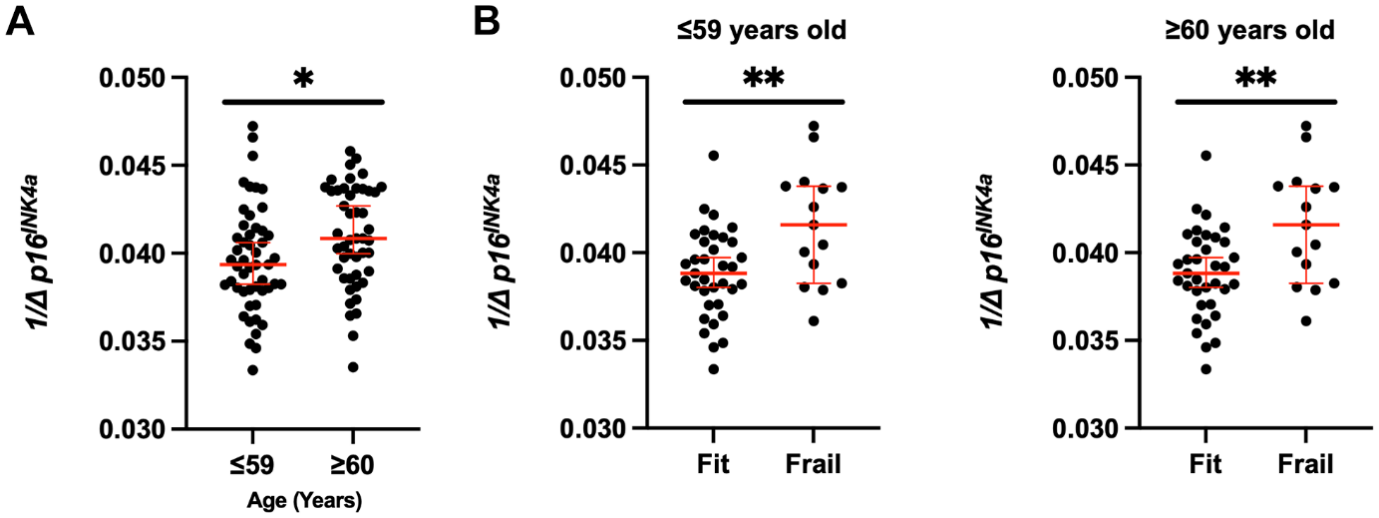
**Increased senescent cell burden in frailty across the ages.** (**A**) *p16^INK4a^* expression was stratified by fit and frail status in all groups (BC and HCT) and by (**B**) age (≤59 versus ≥60 years old). Expression was normalized to 18S. Values represent the median with 95% confidence interval. Students unpaired two-tailed *t* test. * p<0.05, ** p<0.01.

We assessed expression of the senescence marker *p16^INK4a^* in CD3^+^ peripheral blood mononuclear cells (PBMCs). Expression of *p16^INK4a^* in this cell population has been used a molecular readout for circulating senescent cells burden and biological age. We compared *p16^INK4a^* expression to age, frailty, age, and clinical factors to determine any potential associations. Frail patients had higher *p16^INK4a^* expression with median 0.042 (0.035-0.047) vs. 0.039 (0.033-0.046), p<0.01 (Figure 1A); after adjustment for groups (BC and HCT), p=0.33. Figure 1A shows the *p16^INK4a^* expression per frailty group for the whole study cohort, and Figure 1B, 1C per diagnosis group BC vs. HCT. Expression level was significantly higher for those meeting the following of the five individual Fried criteria for frailty: unintentional weight loss, low physical activity, and weakness; p<0.05 (Table 3). Additionally, *p16^INK4a^* expression was significantly higher in those older than 60 with median 0.041 (0.034-0.046) compared to 18-59 years old 0.039 (0.033-0.047), p=0.03 (Figure 2A). When examining the differences in each of the two age groups 18-59 vs. ≥60 separately across frailty groups, there was a similar but stronger trend of a higher *p16^INK4a^* expression in frail patients who are 18-59 years old (Figure 2B). Supplementary Figure 1 shows the *p16^INK4a^* expression vs. age (panel a) and divided by diagnosis group (BC and HCT) (panel b), and by diagnosis and frailty groups with HCT (panel c) and BC shown separately (panel d).

**Table 3:** *Expression of p16^INK4^^a^* by frailty domains.

**Fried frailty domain**	**Median**	**P-value**
**Unintentional Weight Loss**		0.01
0	0.0400	
1	0.0436	
**Exhaustion**		0.54
0	0.0400	
1	0.0404	
**Physical Activity**		0.04
0	0.0398	
1	0.0427	
**Walking Speed**		0.52
0	0.0400	
1	0.0416	
**Weakness**		0.04
0	0.0397	
1	0.0414	

## DISCUSSION

In this cohort study of hematopoietic cell transplantation and breast cancer survivors, we report high prevalence of pre-frailty and frailty with nearly half of the patients meeting at least one criterion for frailty. This is especially alarming when compared to the prevalence of frailty in the general older adult population around 10% [1]. Frail survivors clearly had worse physical functional performance and lower QOL, highlighting the unfortunate reality that achieving remission often occurred at the expense of ongoing functional decline, ongoing morbidity, and compromising restoration of optimal or even individually meaningful QOL.

Frailty was more prevalent among those older than 60 at the time of enrollment, however, it is important to also note the relatively high presence of frailty among patients in the 18-59 age group at 38%. We identified HCT recipients to be at a significantly higher risk of subsequently becoming frail, with a threefold increased risk in male HCT recipients. During HCT, patients receive high dose conditioning regimen, generally consistent of high dose chemotherapy and/or radiation given over a short period of time that invariably causes significant toxicities and collateral damage to healthy tissues and organs triggering systemic inflammation. Not surprisingly, this subsequently drives accelerated aging in HCT recipients that makes this particular group of survivors highly prone to experiencing physiology and biologic aging [13].

Our study shows the clear correlation between clinical measures of frailty, molecular biomarkers of aging, and declines in meaningful restoration of health after cure of the underlying malignancy. The decline in physical functional performance was observed in the widely clinically rather subjective KPS score assessment, in addition to the objective CSPS score reflecting a slower walking speed, compromised balance, and lower endurance. This provides data supporting the additional implications of KPS as an indirect measure of frailty and suggests CSPS as an objective assessment of functional decline in this population. Similarly, frailty was associated with significant decline in QOL as measured by almost all of the SF-36 PCS and MCS as well as FACT-G and disease specific scores, all reflecting the detrimental impact of frailty on all aspects of daily living, mental health, overall functioning, and quality of life.

Previous work has shown that treatment with chemotherapeutics caused increased *p16^INK4a^* expression in breast cancer patients during treatment [21]. While most prior reports examined the impact of chemotherapeutics on aging during therapy, here we report the clinical utility *p16^INK4a^* expression as a biomarker of aging in this cohort of survivors years after completion of treatment. We noted a higher *p16^INK4a^* expression among frail patients, particularly notable for those meeting the Fried frailty criteria for frailty of unintentional weight loss, low physical activity, and weakness. Additionally, we note a progressively higher *p16^INK4a^* expression among those who are pre-frail and frail compared to the fit patients (data not shown), supporting that pre-frail is a biologically intermediate or precursor state frailty, perhaps serving as a biomarker allowing early interventions before more advanced aging occurs. As expected, older survivors had a higher *p16^INK4a^* expression, with a similar but stronger trend of a higher *p16^INK4a^* expression in younger frail patients compared to those who are older than 60. Future studies could determine if the molecular endpoints are predictive of changes in frailty with recovery.

This study is limited by the somewhat heterogeneous patient population with a smaller samples size precluding the analysis of the cohorts separately, as BC and HCT survivors. The smaller size of the cohort also limits the adjustment of *p16^INK4a^* expression levels by age. There is a strong correlation between female gender and breast cancer diagnosis as well, not allowing examination of the impact of gender in this particular malignancy. Additionally, HCT survivors were enrolled earlier than BC survivors, with median time to enrollment of 3 compared to 7 years which could account for some of the differences in frailty observed.

Notwithstanding those limitations, this pilot study demonstrates the alarmingly high prevalence of frailty in two growing cohorts of survivors, those with BC and those undergoing HCT, with detrimental effects of physical functioning, quality of life and overall wellbeing. As a community, this highlights the unmet needs to further understand the steadfast and longitudinal driving mechanisms of accelerated aging in this population. The ultimate goal is to ameliorate or prevent to the extent possible such processes leading to increased short- and long-term morbidities, additional chronic health conditions, and late effects. Our data showing the association between frailty and the senescence marker *p16^INK4a^*, a molecular biomarker for biological aging, years after completion of treatment and further supports considering well-designed senolytic trials in cancer survivors [9]. By targeting the frailty and accelerated aging pandemic in our survivors, we can deliver on the ultimate goal of achieving remission with decreased health care burden and maintaining a meaningful quality of life.

## Supplementary Material

Supplementary Figure 1
