# Supplementary material for: Frailty and pre-frailty associated with long-term diminished physical performance and quality of life in breast cancer and hematopoietic cell transplant survivors
Source: Aging (Albany NY). 2024 Sep 26;16(18):12432–42. doi: 10.18632/aging.206109 (PMC11466481; doi:10.18632/aging.206109)
Supplement: Supplementary Figure 1 [file aging-16-206109-s001.pdf]

SUPPLEMENTARY FIGURE

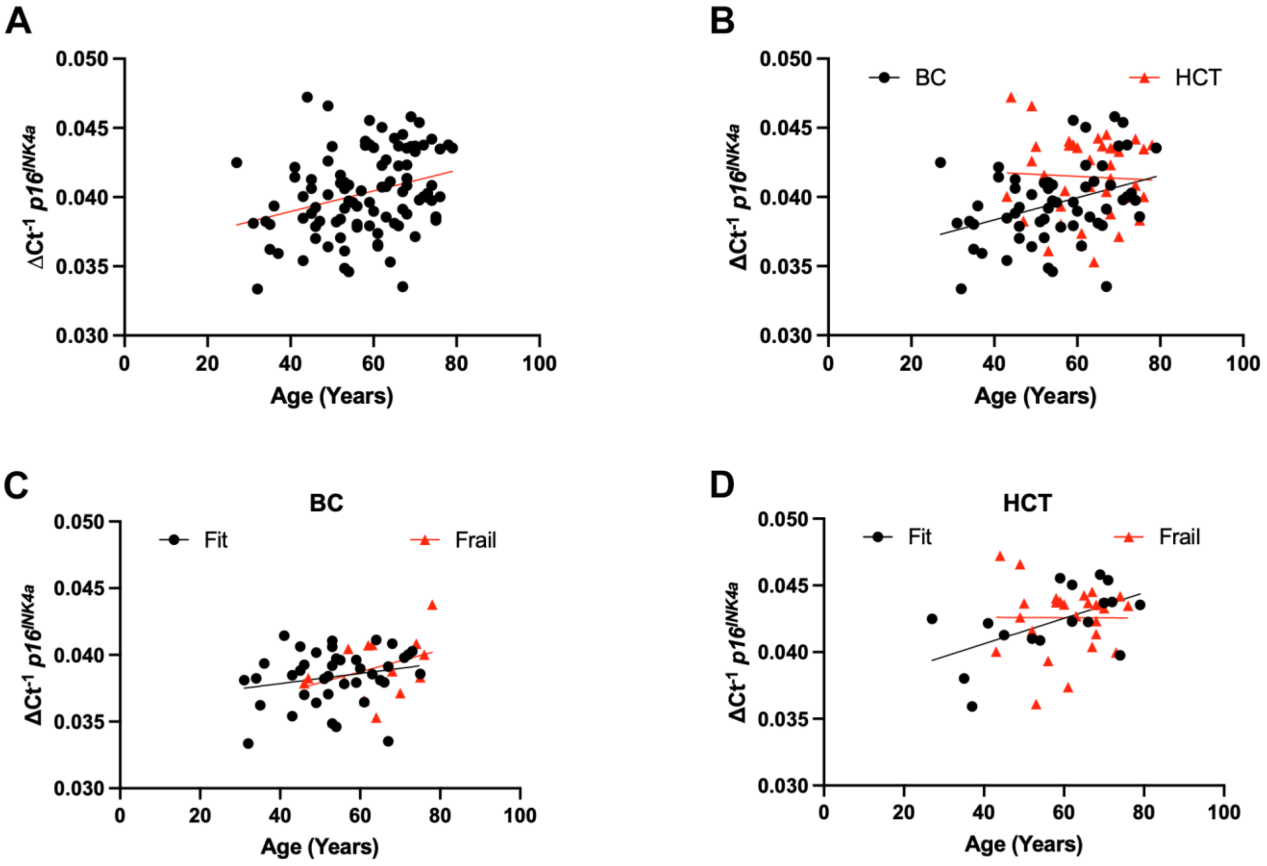

Supplementary Figure 1. Analysis of *p16^{INK4a}* expression with age and disease. (**A**) *p16^{INK4a}* expression was plotted against age for all subjects and (**B**) individually for breast cancer (BC) survivors or hematologic malignancy patients treated with allogeneic hematopoietic cell transplantation (HCT). (**C**, **D**) *p16^{INK4a}* expression was also analyzed with respect to frailty status.
